# Underreported Human Exposure to Mycotoxins: The Case of South Africa

**DOI:** 10.3390/foods11172714

**Published:** 2022-09-05

**Authors:** Queenta Ngum Nji, Olubukola Oluranti Babalola, Nancy Nleya, Mulunda Mwanza

**Affiliations:** 1 Food Security and Safety Focus Area, Faculty of Natural and Agricultural Sciences, North-West University, Private Bag X2046, Mmabatho 2735, South Africa; 2 Department of Animal Health, Faculty of Natural and Agricultural Sciences, North-West University, Private Bag X2046, Mmabatho 2735, South Africa

**Keywords:** aflatoxins, fumonisins, maize, carryover, mycotoxins, rural community, South Africa

## Abstract

South Africa (SA) is a leading exporter of maize in Africa. The commercial maize farming sector contributes to about 85% of the overall maize produced. More than 33% of South Africa’s population live in rural settlements, and their livelihoods depend entirely on subsistence farming. The subsistence farming system promotes fungal growth and mycotoxin production. This review aims to investigate the exposure levels of the rural population of South Africa to dietary mycotoxins contrary to several reports issued concerning the safety of South African maize. A systematic search was conducted using Google Scholar. Maize is a staple food in South Africa and consumption rates in rural and urban communities are different, for instance, intake may be 1–2 kg/person/day and 400 g/person/day, respectively. Commercial and subsistence maize farming techniques are different. There exist differences influencing the composition of mycotoxins in food commodities from both sectors. Depending on the levels of contamination, dietary exposure of South Africans to mycotoxins is evident in the high levels of fumonisins (FBs) that have been detected in SA home-grown maize. Other potential sources of exposure to mycotoxins, such as carryover effects from animal products and processed foods, were reviewed. The combined effects between FBs and aflatoxins (AFs) have been reported in humans/animals and should not be ignored, as sporadic breakouts of aflatoxicosis have been reported in South Africa. These reports are not a true representation of the entire country as reports from the subsistence-farming rural communities show high incidence of maize contaminated with both AFs and FBs. While commercial farmers and exporters have all the resources needed to perform laboratory analyses of maize products, the greater challenge in combatting mycotoxin exposure is encountered in rural communities with predominantly subsistence farming systems, where conventional food surveillance is lacking.

## 1. Introduction

Agriculture is one of the most important contributors to the livelihoods of rural populations in developing countries. In sub-Saharan Africa for example, maize is the staple food and is cultivated by both commercial and subsistence farmers. South Africa has a dual agricultural system, comprising of an advanced commercial farming sector alongside a small-scale subsistence farming sector. Small-scale farmers are mostly present in rural settlements. According to developmental data from the World Bank, South Africa’s rural population accounts for about 33% of the total population [1]. Small-scale farming has long been recognised by South African policymakers and stakeholders as the means through which poverty alleviation and rural development can be achieved [2,3]. In South Africa, about 85% of maize is cultivated in the commercial sector [4], whereas roughly 15% comes from the subsistence farming sector. Average yields of 1.3 and 4.6 tons per hectare for subsistence farmers and commercial farmers, respectively, were recorded between 2008 and 2012 [4]. Commercial farmers implement strategies to reduce losses such as the proper application of insecticides and fungicides, establishment of irrigation schemes, proper harvesting, transport practices and use of suitable storage facilities, among others. On the contrary, subsistence farmers do not have the required resources and skills to ensure the production of quality grains from planting in the field through consumption. Therefore, maize produced by subsistence farmers is often affected by pre- and post-harvest damage, such as fungal infection.

Food security is a serious global issue and continues to top development agendas of most countries, especially in Africa. Food security in SA is still a national crisis due to the high rates of unemployment, poverty, HIV/AIDS, rising food and fuel prices, the recent COVID-19 pandemic, and the recent occurrence of floods in KZN [5]. The COVID-19 pandemic collapsed food production and distribution systems and led to severe food insecurity. Food aids or donations from different organisations were given to South Africans during the pandemic. Sources of the food items and their levels of mycotoxin contamination were unknown. This situation could predispose the population to mycotoxin contamination if the food items came from a mycotoxin endemic region or country.

Fungi are natural contaminants of cultivated products such as cereals and produce secondary metabolites known as mycotoxins [6]. Mycotoxin toxicity occurs at very low concentrations; hence, sensitive and reliable methods are needed for their detection. Once the mycotoxin concentrations are known, chances of the population consuming highly contaminated food can be reduced. There are several methods used to detect mycotoxins in food samples. Therefore, choosing an analytical technique is key in obtaining accurate data, hence, the correct or actual incidence of occurrence of mycotoxins in a sample is a function of the sensitivity of the analytical method used. For instance, previously, 25% of the world’s crop was reported to be contaminated by mycotoxins [7]; recently, improvements in analytical techniques have painted a different picture altogether, with higher contamination values (60–80%) [8]. The cost of analytical services to monitor levels of mycotoxins in food is a substantial part of the total cost of mycotoxin management process. The high costs associated with mycotoxin management may hinder the adoption of interventions by subsistence farmers [9]. This makes it difficult for small-scale farmers to afford these services, even the basic screening techniques.

Fungal development and mycotoxin production are climate-sensitive and SA’s climate is rapidly changing, in step with overall global patterns. Frequent droughts and low rainfall are common traits of South Africa’s climate, which enable favourable conditions for the production of mycotoxins, especially aflatoxins (AFs), produced by members of the genus *Aspergillus.* Common mycotoxin-producing fungal genera *Aspergillus*, *Fusarium,* and *Penicillium* have been isolated in South African maize in varying amounts [10,11,12]. Most studies on mycotoxins, especially on maize in SA, have been conducted in the commercial sector, which is not representative of the entire maize production of the country. More than 95% of the research conducted on South African maize has focused on mycotoxins produced by the genus *Fusarium*, namely fumonisin (FB) and zearalenone (ZEA), despite the reported presence of *Aspergillus* species, especially among small-scale crop producers [13,14,15,16]. Cases of human oesophageal cancer were reported in the Eastern Cape in 1991 [17]. Before then, fumonisin B1 (FB1) had been reported in home-grown maize in January 1990 [18]. These results prompted researchers in SA to focus on *Fusarium* mycotoxins. From review, major mycotoxin reports from both the subsistence and commercial farming systems show no significant different except for AFs, where a high incidence of AFs were discovered in maize and other foodstuff from small-scale compared to commercial farms [19]. This indicates that millions of South Africans from rural settlements are exposed to the effects of mycotoxins on a regular basis. Hence, there is a need to assess South Africans’ exposure to mycotoxins, paying particular attention to AFs within the rural population. Furthermore, the possibility of the co-occurrence of *Aspergillus*, and *Fusarium* species with their mycotoxins, alongside other fungi such as *Alternaria* species and their mycotoxins, exists [15,20,21]. These mycotoxins may act synergistically and are hazards that should not be ignored. Most studies on maize are conducted as multi-mycotoxins research. Some of these have revealed the absence of contamination with aflatoxins (AFs) in SA commercial maize [3,22,23], while others have reported their presence [12,13,16,19,24,25,26,27,28]. Subsistence farmers in SA and their immediate communities are exposed to dietary AFs in maize, milk, carry-over in animal products, and other processed foods daily. This review seeks to highlight the fact that South Africans’ exposure to the effects of mycotoxins is under-reported from the perspective of AFs in foods from subsistence farming.

## 2. Materials and Methods

Preferred Reporting Items for Systematic Reviews and Meta-Analyses (PRISMA) guidelines were used in conducting this literature review [29] to gather information on maize contamination, foods, and feeds with mycotoxins in SA. Google Scholar was used to perform a literature search and keywords and phrases used to extract peer-reviewed studies on mycotoxins conducted in SA. Key words and phrases used to access information were: mycotoxin; aflatoxin; maize; milk; food; feed; SA; cereals; grains; subsistence farming; rural population; Gauteng Province (GP); Eastern Cape (EC); Northern Cape (NC); Kwazulu-Natal (KZN); Mpumalanga (MP); Limpopo (LP); North West (NW); Western Cape (WC); and Free State (FS). One hundred and thirty-nine articles with information related to this review were analysed.

## 3. Possible Sources of Mycotoxin Exposure to Humans and Animals

Over five billion people in sub-Saharan Africa and certain parts of Asia face regular exposure to mycotoxins through contaminated agricultural commodities and air, some as early as during gestation, and could last a lifetime [30]. Currently, between 300 to 400 mycotoxins have been recognised, some of which have been identified as major public health and agro-economic concerns. Mycotoxins identified include citrinin, ochratoxin, patulin, trichothecenes, aflatoxins, zearalenone, nivalenol/deoxynivalenol, fumonisins and ergotamine [17,31,32]. Humans are exposed to these mycotoxins directly through the consumption of contaminated crops or processed food products, or indirectly through foods from animal origin such as tissues, eggs, milk, and other dairy products of animals fed with mycotoxin-contaminated feeds [33]. Mycotoxin carryover from foods of animal origin pose numerous challenges due to the food constituents that regularly contain unquantified amounts of mycotoxins. There is a dearth in information on mycotoxin contamination carryover studies in SA. Moreover, challenges in estimating the consumption patterns of individuals vary from one household to another within a given community [30,34]. Continuous consumption of undiversified diets commonly contaminated with mycotoxins such as maize, peanuts and dairy products, which is the case in SA [35], might cause chronic or acute mycotoxicosis.

### 3.1. Exposure to Mycotoxins through Maize

Maize is one of the major produced world cereal grains after wheat and rice. South Africa is the ninth largest exporter of maize in the world, and is therefore a leading exporter in Africa. In 2018, SA exported maize to 75 countries around the world [36]. Mycotoxins are common contaminants of cereal grains, maize being no exception. In SA, maize is grown in two production systems, commercial and subsistence. South Africa, unlike many other sub-Saharan countries, has an advanced commercial agricultural industry and supplies a sophisticated food market. Contamination of maize with mycotoxins (especially AFs) is generally assumed to not be an issue in SA, as farming is mostly commercial and several studies on mycotoxins substantiate this assumption [22,25]. Strategies to reduce losses due to mycotoxin contamination are usually implemented by commercial farmers. Small-scale farmers rely on natural resources such as rainfall and soil fertility, and lack the required resources to ensure quality maize production. This implies that maize produced by subsistence farmers is at a higher risk of mycotoxin contamination due to poor food production systems and drying and storage methods, which encourage fungal growth and occurrence of mycotoxins [37]. Home-grown maize from parts of northern SA has shown signs of contamination with fumonisins and aflatoxins [15,16]. Generally, the presence of mycotoxins in maize, maize products, and other food products have been confirmed from both farming sectors in South Africa (Table 1, Table 2 and Table 3).

**Figure 1:**
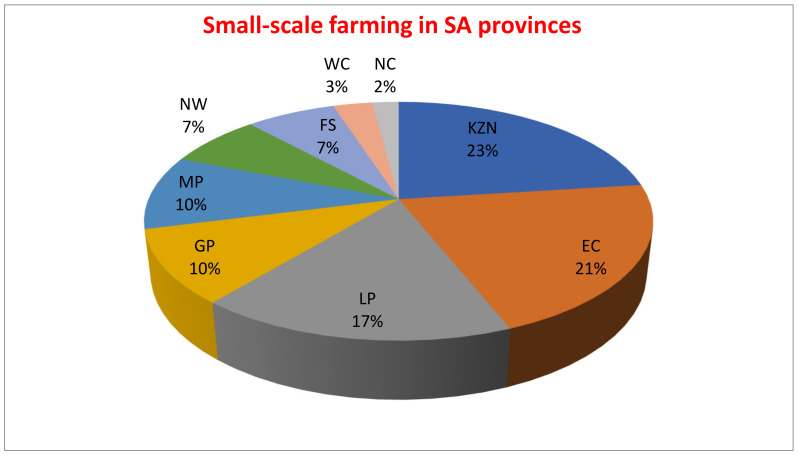
Small-scale farming at provincial levels in SA. Key: NW = North West, LP = Limpopo Province, EC = Eastern Cape, GP = Guateng Province, KZN = KwaZulu-Natal, MP = Mpumalanga Province, FS = Free State, WC = Western Cape, Northern Cape.

There is a growing concern of rural communities’ exposure to mycotoxins in SA through unreported dietary intake, as most households rely on subsistence farming. For example, in KZN, EC, and LP, 23%, 21% and 17% of households, respectively, rely on subsistence farming for their livelihoods [38] (Figure 1). With maize as a staple food, Shephard et al. [39], estimate that SA’s average maize intake is as high as 400 g/person/day. However, this value is different within rural communities, as some household consumption may reach 1–2 kg/person/day [35]. The quality of maize consumed goes a long way to determine the quality of life. Thus, consumers’ exposure to mycotoxins is potentially higher in rural communities when compared to those in cities and towns due to higher levels of maize intake, and higher levels of dietary mycotoxin loads. In addition, subsistence farming practices and pre-harvest (improper management of residue or debris from previous harvests, which contain fungal spores and can increase systemic infection, inappropriate cultivar and use of fertiliser) and post-harvest (unconventional and unhygienic drying techniques as well as traditional or crude storage techniques) handling of crops provide conducive environments for fungal development and mycotoxin production [40].

**Table 1:** Mycotoxin contamination of cereals from subsistence sector.

Type of Mycotoxins	Analytical Methods	Number of Samples (n)	Contamination Rate (%)	Contamination Range (µg/kg)	Median (µg/kg)	References
AF_Tot_	HPLC	50	27	0.080–9.34	4.63	[19]
FB_Tot_			80	12.4–1652.9	906.2	
OTA			56	0.2–51.3	39.2	
ZEA			98	3.6–19.44	8.6	
AFM_1_	HPLC, TLC, ELISA	50	68	5–120	39	[41]
FB_Tot_	HPLC	166	N/A	≥1000	N/A	[42]
AFB_1_	LC-MS/MS	114	47	1–149	N/A	[16]
FB_1_			92	11–18,924	N/A	
FB_Tot_	ELISA	261	88	LOD-21.8	N/A	[43]
FB_Tot_	ELISA	325	89	LOD-21800	1400	[13]
AF_Tot_			100	LOD-49000	9000	
AF_Tot_	ELISA	20	81	N/A	N/A	[44]
FB_Tot_			100	N/A	N/A	
FB_Tot_	LC-MS/MS	400	75	1.8–142,800	N/A	[45]
FB_Tot_	HPLC	211	32	N/A	2150	[14]
FB_Tot_	HPLC-MS	45	67	LOD-16717	2542	[46]
DON			71	LOD-4731	1031	
ZEA			33	LOD-67	34	

**Table 2:** Mycotoxin contamination of maize from commercial sector.

Type of Mycotoxins		Analytical Methods	Number of Samples (n)	Contamination Rate (%)	Contamination Range (µg/kg)	Median (µg/kg)	References
AF_Tot_	Maize	LCMS	282	9.6	LOD-14	N/A	[25]
	Other cereals		63	6.4	LOD-26	N/A	
	Maize silage		109	0	0	0	
	Finished feed		310	5.8	LOD-232	N/A	
FB_Tot_	Maize	LCMS	281	80.1	LOD-16932	177	
	Other cereals		62	19.4	LOD-1119	N/A	
	Maize silage		109	39.8	LOD-1402	N/A	
	Finished feed		310	83.3	LOD-7578	N/A	
DON	Maize	LCMS	314	80.6	LOD-9176	290	
	Other cereals		63	73	LOD-11022	284	
	Maize silage		109	68.8	LOD-2943	122	
	Finished feed		311	67.2	LOD-9805	170	
ZEA	Maize	LCMS	308	47.1	LOD-6276	N/A	
	Other cereals		62	35.5	LOD-195	N/A	
	Maize silage		102	56.9	LOD-3975	2.0	
	Finished feed		301	57.5	LOD-386	5.5	
OTA	Maize	LCMS	269	7.4	LOD-95	N/A	
	Other cereals		51	43.1	LOD-27	N/A	
	Maize silage		101	1	LOD-1.3	N/A	
	Finished feed		259	3.1	LOD-6	N/A	
DON	Wheat	LC-MS/MS	40	12	LOD-593	279.3	[22]
FB_Tot_	Commercial Maize		350	50	LOD-3913	577	
DON				45	LOD-9736	575	
ZEA				7	LOD-354	N/A	
AF_Tot_				N/D	N/D	N/D	

**Table 3:** Mycotoxin contamination of other foods/feeds from subsistence sector.

Type of Mycotoxins	Commodity	Analytical Methods	Number of Samples (n)	Contamination Rate (%)	Contamination Range	Median	References
AFB1	Barley and malt	VICAM	48	91	LOD-4.4		[47]
DON				91	446–1218		
Ochratoxin				80	LOD-1.8		
ZEA				33	132–157		
AF_Tot_	Dog feed	HPLC, ELISA	124	100	LOQ-4946	N/A	[48]
AF_Tot_	Dog food	HPLC	60	87	1.2–352.7	248.3	[49]
FB_Tot_				98	5.2–4653.8	51,556	
OTA				68	0.5–53.6	13.7	
ZEA				96	2.5–2351.4	354.1	
AF_Tot_	Cotton seed for feed	HPLC, VICAM	400	100	24–164	N/A	[50]
AF_Tot_	Ginger from small scale farms	HPLC/ELISA	100	100	3.63–411.1	N/A	[51]
OTA				100	0.9–3.39	N/A	
AF_Tot_	Groundnut from small scale farms	ELISA	46	≥70	LOD-160	N/A	[52]
FB_1_	Muthi (herbal medicines)	HPLC	16	81	14–139	N/A	[53]
AFB_1_	Locally processed maize product (*Ogiri* and *mahewu*)	LC-MS/MS	176	50	N/A	N/A	[23]
FB_1_				37	42–326	N/A	
DON				73	18–32	N/A	
FB_1_	Home-brewed beer from maize (*Umqombothi*)	LC-MS/MS	N/A	53	LOD-182	N/A	[54]
DON				84	N/A	N/A	
AF_Tot_	Home-brewed beer	HPLC/TLC	29	28	200–400	N/A	[55]
ZEA				45	3–2340	N/A	

Key: AF_Tot_ = Total aflatoxins, AFB_1_ = aflatoxin B_1_, AFM_1_ = aflatoxin M_1_, DON = deoxynivalenol, FB_Tot_ = Total fumonisin, FB_1_ = Fumonisin B_1_, ZEA = zearalenone, OTA = ochratoxin A, LOD = Limit of detection, ELISA = enzyme linked immunosorbent assay, TLC = thin layer chromatography, HPLC = high performance liquid chromatography, LC-MS/MS = liquid chromatography tandem mass spectrometry.

Mycotoxins commonly found and reported in foods and feeds across SA were FBs, AFs, ZEA, DON and OTA, among others (Table 1, Table 2 and Table 3). South Africa mycotoxin data currently available are not sufficient to draw absolute conclusion that SA maize is safe from AF, in particular [22,25]. The high prevalence of mycotoxins in SA can be attributed to climatic-related stresses, such as drought, flooding, elevated CO_2,_ and extreme temperatures, predispose maize plants to fungal infection [56]. Rheeder et al. [14] reported that drought conditions specifically increase levels of FB in maize in SA. Studies elsewhere have revealed the predominant role of drought and high temperatures in elevated AF production in maize [57,58]. The occurrence of drought is common in the NW and the FS provinces, which are main maize-producing provinces in SA, stresses the crop thus increasing the risk of higher levels of FBs and AFs in maize. Flooding before the maize harvest in Argentina resulted in more than 65% of samples tested being above the mycotoxin risk threshold [59]. Jakšić et al. [60] exclusively reported the presence of aflatoxigenic species *A. flavus* at post-flood locations. Since water is a major promoter of fungal proliferation [61], the expected aftermath of flooding is even higher, as the occurrence of flood creates a moist environment conducive for fungal growth and mycotoxin production. In SA, flooding is a common phenomenon in KZN, explaining the high prevalence of mycotoxins from the subsistence sector in this province [13]. In all, favourable climatic conditions for the production of mycotoxins in food have been reported in most SA provinces, of which five have been declared drought disaster provinces [56]. This implies that these populations are at a risk of exposure to dietary mycotoxins, which is proportional to the percentage of maize consumed from the subsistence sector. While maize produced in the commercial sector is mainly for export, produce from small-scale farmers is mainly for household consumption and the excess is sold to the immediate community. With the high consumption rates in rural communities and poor storage facilities, there is a higher probability of contamination with mycotoxins, which are often underreported. Most subsistence farmers in SA cultivate their fields with seeds from a preceding harvest [13], increasing the danger of systemic infection with plant pathogens. It has been reported that monoculture and late planting increase fungal inoculum and pest damage, leading to increased fungal infection in crops [62]. The quantity of maize produced determines the specific storage practices to be employed. Storage units used by farmers in SA rural communities do not promote proper drying of maize, increasing interaction with insects and promote fungal infection and production of mycotoxins [63,64]. Commercially produced South African maize has been reported to be AF free [22] and or AF levels are well within regulatory levels [25]; however, improper harvest and storage practices by subsistence farmers can favour the growth of fungi, resulting in high levels of AF contamination in maize from this sector.

### 3.2. Exposure to Mycotoxins from Other Food Sources

Processed foods, such as traditional fermented beverages produced from cereals, are widely consumed in Africa. Cereals grown in Africa are often contaminated with multiple mycotoxins, and knowledge about the carryover of various mycotoxins from cereals to beverages is scarce. Medina and co-workers reported on food processing techniques being inadequate to completely eliminate some mycotoxins from food and feed as a result of their heat and chemical stability, which permit them to withstand the rigours of processing [65,66]. For instance, *Oshikundu*, a popular non-alcoholic sorghum fermented beverage in Namibia, was analysed for fungal metabolites and their fate during processing, revealing that the transfer rates of mycotoxins from cereals to *oshikundu* exceeded 50% [67]. *Aspergillus* metabolites were the most common and included aflatoxins, cyclopiazonic acid, and 3-Nitropropionic acid. *Fusarium*, *Penicillium*, *Alternaria* and *Claviceps* mycotoxins were also detected. There are different types of beers brewed from cereals in South Africa, and for socio-cultural reasons, mouldy maize is used intentionally in the Eastern Cape and Limpopo to brew beer as it is believed to improve flavour [68,69]. The cereals used are mostly from subsistence farming, which are most often highly contaminated with mycotoxins as a result of poor pre- and post-harvest handling [40]. The fates of most locally brewed beers with regard to mycotoxin content in SA are yet to be determined. However, it is possible to assume that the transfer rate of mycotoxins might be similar to the 50% contamination rate of the *oshikundu* beer in Namibia, due to the mouldy raw materials derived from the subsistence farming sector.

Most mycotoxins (example AFM_1_, AFB_1_) can withstand the rigours of food processing due to their heat and chemical stability. Aflatoxin M_1_ is not affected by regular processing, as is evidenced by AFM_1_ which was reported to be higher in cheese than in raw milk [70]. These results are similar to those reported by [71], where the occurrence and concentration of AFM_1_ was almost six times higher in cheese compared to raw milk. This is substantiated by reports of high incidences of AFM_1_ in processed cheese in Egypt [72]. Anelli et al. [73] reported similar results for Cave cheese. At the time of this review, data on mycotoxin contamination of cheese produced in SA were unavailable.

### 3.3. Exposure to Mycotoxins through Carryover-Effects of Farm Animals

Animals are exposed to various mycotoxins that may be present in feed, such as aflatoxins, fumonisins, zearalenone, deoxynivalenol and ochratoxins, among others. Mycotoxins occur in protein-rich concentrates, cereal grains, corn gluten, soybean products, and pressed cakes from oil plants, such as peanuts, sunflower seeds, cotton seeds, palm kernels and copra; which are feed constituents. When a high percentage of contaminated protein-rich concentrates are incorporated into the diet, it is the main source of mycotoxins in the animal [74]. The possibility exists that mould contamination might alter the composition and activity of rumen microorganisms. Aflatoxin B_1_ consumption in feed by lactating animals result in its metabolism either into aflatoxicol, a metabolite 18 times less toxic than AFB_1_ [20,75], or is absorbed in the digestive tract and hydroxylated in the liver forming AFM_1_ that appears in blood, urine, and is also excreted in milk [76]. Unlike monogastric species, ruminants are less susceptible to the effects of mycotoxins, as the ruminal fluid microbiota in the digestive system helps to degrade the mycotoxins. However, the capacity of the rumen to detoxify mycotoxins can be limited, resulting in the accumulation of AFM_1_ in milk [77]. This explains why ruminants seldomly develop mycotoxicoses, as the rumen microbiota usually act as the first line of defence against the mycotoxins. In healthy cattle, up to 12 mg/kg of OTA can be inactivated when ingested alongside the feed. OTA is readily converted into a less toxic OTα by the rumen microbiota, and minute amounts of OTA are absorbed [74]. On the contrary, non-ruminants, such as pigs, are the most sensitive to OTA (Table 4).

Zebib, Abate [78] revealed that all milk samples collected among value chain actors were contaminated with AFM_1_. Aflatoxin M_1_ is detectable within 12 h in milk after an animal consumes feed contaminated with AFB_1_ [79]. Conversion of AFB_1_ to AFM_1_ is through hydroxylation of the ring of the difuranocoumarin tertiary carbon. The -OH group increases its solubility in water, and allows for rapid excretion in faeces, urine, and milk. However, the extent of carryover is also influenced by nutritional and physiological factors, including feeding regimens, rate of ingestion, rate of digestion, the health of the animal, capacity of hepatic biotransformation, farming systems, seasons, geographic location and environmental conditions. For example, studies have reported that milk and dairy products produced in warm seasons are less contaminated than those produced in cold seasons [80,81,82]. However, transformation rates vary depending on the species of animal and their health status. Data on meat and meat product contamination by mycotoxins also differ depending on the animal species involved. The content of mycotoxins in edible tissues from bovine species is relatively low, since mycotoxins are partly degraded in the rumen, rapidly metabolised in the liver, and consequently do not contribute significantly to human exposure [77]. Fish from aquaculture are fed with different feed and raw materials, which have been reported to be contaminated with mycotoxins. However, fish represent the least studied animal concerning mycotoxin occurrence when compared to other animal-derived products [83].

Apart from AFs, OTA is a mycotoxin that has been investigated concerning carryover, which often co-occurs with its analogue toxin OTB, which is a non-chlorinated minor toxic metabolite. Contamination with ochratoxin-producing fungi has been observed globally and involves foodstuffs such as grapes, wine, fruits, cereals, coffee, cocoa, edible nuts, pulses, beer, and spices. The level of OTA in milk not converted to OTα is small compared to the levels of OTA contamination of grains observed during daily feeding practices [84]. This is enough to cause significant danger to consumers.

Deoxynivalenol (DON) is a naturally occurring mycotoxin with strong emetic effects after consumption, and is therefore also known as vomitoxin. The susceptibility of ruminants to DON is low, as DON is completely converted into the less toxic DOM (the de-epoxidised metabolite of DON) in the rumen. DON is of economic concern mainly due to its neurotoxic effects, resulting in severe depression and low animal productivity. However, no human diseases as a result of carryover have been recorded. Monogastric animals, such as pigs, are the most affected by DON exposure (See Table 4).

Zearalenone is converted by rumen microbiota to hydroxy-metabolite α–zearalenol at a rate of 90%, with higher oestrogenic effects compared to its parent ZEA. It has a lower rate of absorption in the liver, accounting for the low susceptibility of dairy cattle [85,86,87]. Zearalenone and its metabolites can be excreted with milk in minute amounts, usually below significantly quantifiable levels [87].

Fumonisin is a common contaminant of maize and maize products. There exists limited information on its biotransformation and carryover. Even at high concentrations of FBs in feed, low carryover from feed into milk has been reported for bovine species [77].

Patulin mainly occurs in damaged fruit, fruit juice and vegetables, and is periodically considered as a co-contaminant in by-products intended for animal feed [88]. Patulin is metabolised in the liver and its elimination pathways include faeces and urine, with the majority of the toxin being excreted within 24 h. A carryover of 2–3% is possible in soft tissue and blood [84].

The T-2 toxins (trichothecene) carryover in milk is possible, with levels ranging between 0.5–2.0% [74].

**Table 4:** Animal exposure to mycotoxins and rate of mycotoxin carryover in animal products.

Mycotoxin	Main Product of Rumen Metabolism	Carry-Over Product	Carryover (μg/kg)	References
Aflatoxin B_1_	AFB_1_	Meat products, such as liver sausages	0.89–1.69	[84,89,90,91,92,93,94]
		Meat	0.30–52.93	
		Dried meat	105.4	
		Eggs	0.10	
		Yolk	0.22	
		Albumen	0.27	
		Quail liver	0.19	
		Sea bass fish	0.02	
	Aflatoxicol	Milk	0–12.4	
	AFM1	Milk	3–9	
Cyclopiazonic acid	Unchanged	Milk	0.4–0.7	[95]
Fumonisin B_1_	Unchanged	Chicken liver and muscle	0.79–44.7	[84,96]
		Turkey liver and muscle	1.41–41.47	
		Milk	0.16	
		Porcine liver and kidney	2	
Ochratoxin A	Ochratoxin-α	Milk	0.02	[20,84,97,98,99,100]
		Cheese	0.07–0.11	
		Porcine offal	0.17–0.20	
		Fermented sausages and hams	6.87–7.83	
		Beef kidney	2.73–4.43	
		Beef liver	1.71–2.13	
		Chicken muscle	4.7	
T-2 toxin	Unchanged	Milk	0.06	[74,84,101]
		Chicken tissue	3.71–3.93	
DON	De-epoxy-DON (DOM)	Milk	0.13 (cows)	[84,102,103]
		Milk	0.01 (dairy ewes)	
		Porcine bile	668	
		Porcine kidney	100.2	
		Porcine liver	33.4	
		Porcine serum	15.36	
		Porcine muscle	10.69	
		Porcine fat	1.34	
		Turkey bile	0.01	
		Salmon fish fillet	18.6	
Zearalenone	α-zearalenol	Milk	12.91	[104,105,106]
		Porcine liver	1.60–17.77	
		Porcine spleen	6.46–47.81	
		Chicken liver	5.10	
Patulin	Unchanged	Milk	0.8	[84]
Rye ergot	Unchanged	Poultry	0.01	[74]

Several studies have been conducted on the effects of carryover, but such studies are limited in SA, and the unavailability of data does not necessarily imply mycotoxin exposure through carryover does not exist. Phokane et al. [64] reported that most subsistence farmers used mouldy and damaged maize as animal feed. The increasing consumption of animal products such as dairy, from different animal species, emphasises the need for carryover effect data from these animals as well, to ensure food safety. In Ethiopia, for example, all milk samples were reportedly contaminated with AFM_1_ in varying concentrations [78]. There is a need to extend AF carryover surveillance to other mycotoxins and precursor compounds such as sterigmacystein and minor metabolites such as aflatoxicol.

## 4. Co-Occurrence of Mycotoxins

Different fungal species can grow in the same plant causing the co-occurrence of multiple mycotoxins [107]. Moreover, composite feed is made up of a mixture of several raw ingredients, exposing it to contamination with multiple mycotoxins [108,109]. The frequent co-occurrence of mycotoxins amplifies the health risk they pose and varies with the health status of individuals. With regard to co-occurrence, Tolosa et al. [20] reported that nearly 65% of analysed samples contained at least two mycotoxins, with AFB1 and FBs being the most observed in the finished feed and maize from sub-Saharan Africa, South and Southeast Asia and Oceania. Kamala et al. [110] reported similar results, with a 69% co-occurrence of AFs and FBs in maize samples. Elsewhere, Stanciu et al. [111] investigated 66 samples of wheat grains and flour, for the occurrence and co-occurrence of different mycotoxins, and found co-occurrence of between two and five mycotoxins in more than 40% of the samples. Based on the observations from these studies, the recurrent co-occurrence of mycotoxins in food and feed implies that a generally applicable exposure assessment is not feasible, and that there is paucity of data on exposure to multiple mycotoxins to quantifiable markers. This is due to the following reasons: firstly, the complexity in determining the consumption patterns of different foods known to be susceptible to mycotoxins including cereal, cheese, milk, juice and homebrewed beer in SA. Secondly, the scarcity of data on contamination with mycotoxins of foods that is commonly consumed in SA. Food items, such as milk and cheese, which are highly consumed in SA, are known to have high rates of contamination with AFM_1_ elsewhere [78]. Unfortunately, SA might have few data on mycotoxin contamination in these products, as their rates of consumption data are unavailable. Furthermore, pig breeds that are susceptible to OTA, which are highly consumed in South Africa, lack mycotoxin carryover data as well as with other species, such as fish. Thirdly, the frequent co-occurrence of these mycotoxins makes it difficult to attribute some of the health effects to one mycotoxin. The possible development of novel diseases with unknown symptoms also exists. Most health effects of mycotoxins reported are often limited to single mycotoxins, but data on the combined effects of these mycotoxins are scarce [112] and warrant more attention.

## 5. Health Effects of Mycotoxins Recorded in South Africa

The World Health Organisation has identified mycotoxin contamination of food as a global food safety issue [113] with subsistence farming communities being the most at risk of exposure. Eliminating mycotoxins from the food supply chain appears impossible due to their thermal and chemical stability [66]. The severity of individual’s ill-health due to mycotoxin exposure depends on the toxicological properties of the particular toxin (acute, long-term toxicity, mutagenicity, teratogenicity and carcinogenicity), age and the extent of the exposure [112]. Low levels of chronic exposure to mycotoxins pose different health risks. Exposure to multiple mycotoxins may result in different signs and symptoms than if exposure was to a single mycotoxin, as earlier mentioned. Mycotoxins are therefore considered important food/feed contaminants, which carry a high health risk in SA [47,114,115]. Health risks associated with consumption of mycotoxin contaminated food/feed are given in Table 5.

Exposure to AFs has resulted in different kinds of aflatoxicosis in humans. Acute aflatoxicosis, often caused by multiple exposures, could result in death in severe cases, while chronic aflatoxicosis may lead to hepatocellular carcinoma, suppression of the immune system and in some cases, stunted growth. The International Agency for Research on Cancer classified AFB_1_ as a Group 1 carcinogen [116]. Reports on outbreaks of massive aflatoxicosis with high rates of mortality have been made globally [34,110,117,118].

**Table 5:** Health effects of common mycotoxins on humans and animals.

Mycotoxins	Health Effects		References
	Humans	Animals	
Aflatoxins (B_1_, B_2_, G_1_, G_2_, M_1_, M_2_)	Liver cancer, hepatocellular carcinoma, stunted growth, jaundice, immunosuppressive	Reduction in weight gain and kidney malfunction in rats, thymic depression in pigs, low feed intake, anorexia and decreased milk production	[77,112,119]
Deoxynivalenol	Vomiting, nausea, diarrhea, anorexia, severe gastro-intestinal (GI) toxicity	Cytotoxicity, diarrhea and anorexia	[119,120,121,122]
Fumonisins (B_1_, B_2_)	Esophageal and liver cancer, neural tube defects	Atherosclerosis in monkeys, leukoencephalomalacia in horses, equines and rabbits, porcine pulmonary edema and pulmonary artery hypertrophy in swine, kidney and liver cancer in rodents, cancer of the esophagus in rats	[112,119,121,123,124,125,126,127,128,129,130,131]
Ochratoxin A	Urothelial tumors, chronic interstitial nephropathy, renal failure, it can cause an adverse effect on the foetus in the womb due its ability to cross the placenta and cause the malformation of the central nervous system and damage the brain	Mycotoxic Porcine Nephropathy (MPE)	[34,119,132,133,134,135]
Patulin	Hemorrhages, ulcerations, vomiting and nausea, gastrointestinal, (GI) disturbances	Include liver, kidney toxicity, spleen damage and toxicity and immune toxicity	[17,119,120]
Rye ergot	Causes ergotism, a human disease known as St. Anthony’s fire; delirious seizures, gangrenous and convulsion	No known health effects	[119,136,137]
T-2 toxin	Alimentary toxic aleukia (ATA) in humans	T-2 toxin induced apoptosis and developmental toxicity in zebrafish embryos	[119,138,139,140]
Zearalenone	Uterine fibroids, pituitary adenomas, Hepatocellular carcinoma, abortion, ZEA is associated with early puberty in girls, infertility in men, can stimulate breast cancer	Liver damage in mice, nephropathy in rats, hyperestrogenic syndrome in pigs, abortion, causes an increase in the incidence of pituitary tumors and liver cell in mice, causes hyperkeratotic papilloma in rats, involved in the development of tumour in the GI tract, causes hepatocellular adenomas in mice	[112,119,121,125,127,141,142,143,144]

With the increasing consumption of maize, particularly in rural subsistence farming communities in SA where storage infrastructure is poor, an increased risk of mycotoxigenic exposure exists. There are few reports on the health effects of mycotoxins on humans and animals in SA, attributable to low levels of research into understanding the impact of mycotoxins on food safety and their associated health consequences [145]. A few cases have, however, been reported, for example, the South African Medical Research Council has reported aflatoxicoses due to the consumption of large amounts of AFs in peanut butter (272 μg/kg total AF and 165 μg/kg AFB_1_) among primary school children in the Eastern Cape as part of the Primary School Nutrition Programme [146]. Cases of kwashiorkor, marasmus and underweight children had long been reported in Durban in 1992. These findings correlated with impaired liver function. In 2012, a relationship between AFB_1_ and cases of kwashiorkor, marasmus and underweight were reported in SA [67]. Evidence of a link between undernourishment and consumption of mycotoxin contaminated food has been found, and has shown that fungi and mycotoxins have the ability to reduce the nutritive value of food [53,147]. Although the cause of stunted growth cannot be assigned to mycotoxin contamination alone, increased consumption of mycotoxin contaminated food is one of the underlying causes of this health problem [148,149].

Consumption of FB contaminated foods has long been associated with oesophageal cancer, abdominal pain, diarrhoea, stunted growth, hepatotoxicity and nephrotoxicity in rodents, equine leukoencephalomalacia in horses, serious pulmonary oedema and left ventricular dysfunction and hepatotoxicity in pigs [4,116]. The IARC classified FB as Group 2B carcinogens (possibly carcinogenic to humans). Fumonisins have been linked to a high incidence of oesophageal cancer in rural communities in SA, such as in the Eastern Cape and Limpopo provinces, due to the preference of using mouldy kernels to produce traditional *umqombothi* beer, and thereby posing a risk of mycotoxin exposure [68,69]. A correlation was reported between levels of FB1 exposure and patients’ blood and brain lesions at Wentworth Hospital’s Neurosurgical Unit in KZN [150]. FBs have also been implicated in the high incidence of neural tube defects in rural populations known to consume mould-contaminated maize in SA [151,152,153]. South African maize is highly contaminated with mycotoxins, especially home-grown maize including FB (see Table 1). There are high levels of household consumption of maize in rural communities in SA, with intake levels of 1–2 kg/person/day [35,154]. It is safe to say rural communities in SA, whose livelihoods depends on home-grown crops, such as maize and groundnut (crops that are highly susceptible to mycotoxin contamination), are highly exposed to the deleterious effects of mycotoxins.

Occurrence of ZEA in foods is generally low, but its importance rests in its oestrogenic effect in mammals. Zearalenone has been associated with hyperoestrogenic syndromes and is known to be an eliciting factor of early puberty in girls [155]. Even though the ability of ZEA to stimulate the growth of human breast cancer cells has been reported [156], the IARC classified ZEA as Group 3 (not classifiable in terms of carcinogenicity in humans). ZEA is structurally identical to the hormone estradiol and has an affinity for oestrogen receptors, thereby affecting fertility in livestock and humans. Various in vivo oestrogenic potential effects have been reported for zearalenol and its metabolites. In Africa, ZEA have been viewed as a contributory factor in increasing infertility in males [157]. Levels of ZEA up to 426 µg/kg were quantified in cereal-based products in SA [55]. These levels are far above the maximum level of 100 µg/kg stipulated by the European Commission’s [158] regulation of unprocessed cereals.

Occurrence of OTA in cultivated crops is a health concern, as toxicological reports show it is nephrotoxic, carcinogenic, teratogenic, hepatotoxic and immunotoxic. The IARC classified OTA as Group 2B carcinogens (possibly carcinogenic to humans). OTA has been reported in traditional home-brewed beer in KZN in large amounts varying between 876 to 2340 µg/kg [52]. This is a product consumed on a daily basis by the local population, and exposure to such high amounts over prolonged periods of time, could result to chronic health complications.

Contaminated maize grains, which are not fit for human consumption, are usually channelled into feed formulations, where they are widely reported to pose health risks to pets and farm animals [44,67]. All mycotoxins in feed, even at low levels, have a broad spectrum of effects on animal health that include immune dysfunction, digestive disorders, carcinogenicity, neurotoxicity, hepatoxicity, impaired reproduction and even death [4,59]. For example, during an outbreak of animal aflatoxicosis in Gauteng in 2011, more than 220 dogs died due to the consumption of pet food highly contaminated with AFB_1_ (up to 4946 μg/kg) [48]. Similarly, nephropathy in pigs was reported in South Africa, with a combination of OTA and FB_1_ at concentrations varying between 67–251 µg/kg and 5021–5289 µg/kg, respectively, as well as penicillic acid at 149–251 µg/kg [47]. Studies on animals have reported that ZEA, DON, OTA and AFB_1_ seriously affect fertility by damaging the sex organs, gametes and the disruption of steroidogenesis. Ingestion of FB, AFB_1_ and DON in pigs disrupt the intestinal barrier, leading to suppressed immune response, reduced feed intake and poor weight gain [159]. DON consumption has been reported to cause low efficiency of feed conversion in livestock and anorexia in pigs and other monogastric animals [4], while ruminants and poultry seem to be resistant to DON [160]. However, diets containing low levels of DON have been reported to result in lower productivity, impaired immunity, and higher susceptibility to infectious diseases in poultry [160]. ZEA contaminated feeds have been reported to significantly affect metabolic rates of nutrients, activities of serum enzymes, and genital organs in growing-laying hens [161]. Continuous exposure of farm animals to mycotoxin-contaminated feeds can induce clinical signs of depression, anorexia, weakness, weight loss and sudden death [44].

## 6. Conclusions

On a global scale, AFs are the most studied mycotoxins due to their deleterious health effects, with studies dedicated to *Aspergillus* species’ mycotoxins. On the contrary, in SA, most research on mycotoxins has been conducted on *Fusarium* species’ mycotoxins. Reports on FB1 in home-grown maize in the early 1990s were found to correlate with incidences of oesophageal cancer in the Eastern Cape. This explains why the primary reason for most mycotoxins’ research in SA has focused on FBs. Most results on AF contamination of maize in SA come from multi-mycotoxin analyses [22,25]. AFs contamination is similarly very high in rural communities in SA, as it is in other African countries, where farming is mainly subsistence-based. Literature shows that some mycotoxin-producing fungi and their secondary metabolites co-exist under favourable conditions and that there is evidence that AFs act synergistically with FBs in grains [110,162]. Maize samples from LP, KZN and MP were heavily contaminated with both AF and FB [13,16]. Zearalenone and DON regularly co-occur, an observation which may be important to feed safety in view of reported combined effects [25]. The co-occurrence of multiple mycotoxins in maize increases the probability of interactions, resulting in additive or antagonistic effects, which may increase its risks to human health [163]. Thus, an absolute conclusion should not be made with regard to maize safety with respect to mycotoxins, as the contaminated maize produced in the subsistence farming sector is hardly reported. This goes a long way in emphasising the importance of farming methods and practices in mycotoxin mitigation in sub-Saharan Africa. South Africa’s climatic condition, during the pre-harvest production period, and socio-economic factors may favour the growth of mycotoxin-producing fungi, which is a serious issue in most rural districts of SA. Since drought is a common phenomenon in most provinces of South Africa and varies yearly, this situation will increase the risk of contamination of crops with mycotoxins. While commercial farmers and exporters have all the resources needed during pre- and post-harvest, storage facilities and access to laboratory analyses to combat mycotoxins, the greater challenge is in rural, predominantly subsistence-farming communities, where conventional food surveillance is lacking.

South Africa has a dual maize farming system, comprising of an advanced commercial farming sector (85%), alongside the subsistence farming sector (15%). Maize from the commercial sector might have been declared aflatoxin-free [22,25], and the risk of co-occurrence contamination with major mycotoxins (AFs and FBs) could be a possibility. Claims of South African maize being mycotoxin-free are not entirely true due to subsistence-farming exclusion. If policy makers and shareholders in SA recognise small-scale farming as a means to alleviate poverty, there will be a need for government and shareholders to put in place efforts to educate farmers in rural communities on better pre- and post-harvesting practices. Fifteen percent of maize produced by small-scale farmers are consumed by them, with the rest being sold to immediate rural populations, which represent 33% of the country’s population. Thus, millions of South Africans are exposed to the effects of mycotoxins, which goes unreported. There is a need to involve the overall agricultural system in mycotoxin research studies. Lastly, there is a need to avoid socio-cultural practices that will jeopardise the health of people, such as the production of traditional or home-brewed beer from mouldy cereal contaminated with mycotoxins.

## Data Availability

Not applicable.
